# Responses of smoking and nonsmoking cancer patients to drug treatment: A protocol for systematic review and meta-analysis

**DOI:** 10.1097/MD.0000000000022104

**Published:** 2020-09-18

**Authors:** Lishi Wang, Helin Feng, Jing Li, FengXia Liu, Qingyi Liu, Jiafu Ji, Arnold Postlethwaite, Weikuan Gu, Baoen Shan

**Affiliations:** a Department of Basic Medicine, Inner Mongolia Medical University, Jinshan Development Zone, Huhhot, Inner Mongolia, P.R. China; b Department of Orthopedic Surgery and Biomedical Engineering, The University of Tennessee Health Science Center, Memphis, TN, USA; c Research Center, The Fourth Hospital of Hebei Medical University, Shijiazhuang, Hebei; d Beijing Cancer Hospital and Key Laboratory of Carcinogenesis and Translational Research, Department of Gastrointestinal Surgery, Peking University Cancer Hospital and Institute, Beijing, P.R. China; e Department of Medicine, Division of Connective Tissue Diseases, The University of Tennessee Health Science Center; f Research Service, Memphis VA Medical Center, Memphis, TN, USA.

**Keywords:** cancer, hazard ratio, lung, patients, smoking

## Abstract

**Introduction:**

Smoking is well-known to increase cancer risk, particularly risk of lung cancer, and negatively affects efficacy of cancer treatment. However, recent evidence suggests that among cancer patients, paradoxically, smokers respond to treatment better than non-smokers. We propose to conduct a focused review and meta-analysis to compare response to drug treatment between smoking and non-smoking cancer patients.

**Methods and design:**

We will collect data from large clinical trials of therapies for cancer patients which have included smokers and non-smokers. We will search PubMed, PMC/ MEDLINE, SCOPUS, Embase, and the registries for clinical trials and four major clinical journals up to June 30, 2019. Search terms will be “Drug name” phase-3” or “Drug name” phase-III.” Data collection will be focused on randomized clinical trials of cancer drugs that enrolled at least 100 participants and reporting treatment results from smoking and nonsmoking patients. Initial selection criteria will be clinical trial studies of drug treatment of 100 or more cancer patients, and reporting hazard ratios (HR) for smokers and non-smokers. Two persons will be searching such publications independently, or data will be provided, double checked, or confirmed by authors. Multiple sub-group analyses will be conducted by at least two persons to avoid bias or experimental errors.

**Discussion:**

The results will clarify whether smoking and response to treatment of cancer are linked not. Our results may possibly identify drug/s that work better among cancer patients who are smokers.

**Trial registration:**

PROSPERO registration number: CRD42019146402.

## Abstract 2

Supplemental Digital Content is available in the text

## 1 Introduction

Smoking by humans (of various burning plant materials) has a long history dating back ∼5000 years. Tobacco contains several toxicologically significant chemical groups which are known to cause many health problems and increase risk of several diseases.^[1]^ Tobacco can also be deadly for non-smokers via second-hand tobacco smoke that contributes to heart disease, cancer, and other diseases.^[1,2]^

Cancer, by definition, is a group of diseases involving abnormal cell growth with the potential to invade or spread to other parts of the body with potentially lethal consequences, and the incidence of cancer increases with age. Cancer is caused by complicated genetic and environment interactions. While it is affected by genetic background and mutations, it is also affected by many environmental factors such as radiation from ultraviolet rays and toxic substances, such as the chemicals in tobacco smoke. While genetic factors are difficult to alter, environmental factors have been the main targets of cancer prevention.^[3,4]^

Smoking also influences response to cancer treatment.^[5]^ People who continue to smoke during treatment of early stage lung cancer almost double their risk of dying.^[6]^ While most clinical trials on non-small-cell lung cancer (NSCLC) suggest that smoking negatively impacts drug treatment,^[7]^ there are fewer data for this effect than for that of smoking as an accelerant of cancer progression. For example, in a review, Mitchell et al concluded that “The evidence does not support smoking history as a predictor of response to non-EGFR-targeted therapies or chemotherapy.”^[8]^ The impact of smoking on treatment of patients with other types of cancer has not been thoroughly analyzed.

Smoking cessation has been an integral part of lung cancer treatment.^[9,10]^ According to the Centers for Disease Control and Prevention (CDC), the smoking population in the United States has decreased in the past 12 years from 20.9% of every 100 adults in 2005 to 14.0% of every 100 adults in 2017.^[11]^ However, despite great strides in reducing the number of smokers, smokers remain a substantial percentage of cancer patients and in the population worldwide.^[12]^ In the United States currently 14.0% of the population smokes, that is, over 30 million people in the United States are still smokers.^[11]^ Accordingly, smoking will remain a risk for cancer and will negatively impact disease, drug treatment of cancer, in the United States and worldwide.

Knowing that there will be a population of smokers among cancer patients, optimal treatment for such a population should be a priority of treatment research. However, just how and to what extent smoking impacts cancer treatment is not completely clear.^[8,13]^

Reports on treatment of NSCLC with Programmed death-1 (PD-1) or Programmed death-ligand 1 (PD-L1) inhibitors suggest a better response to these treatments in smokers than non-smokers. A recent study using pembrolizumab (a PD-1 inhibitor) as the first-line treatment in an open-label and randomly assigned clinical trial, showed that the HR values of both former smokers and current smokers were l lower than that of the non-smokers.^[14]^ The number of patients in each category (e.g., current smokers, former smokers, and non-smokers) was more than 100. An early report on the treatment of NSCLC with pembrolizumab by Reck et al with a smaller patient population of 305^[15]^ also showed that former and current smokers had lower HR of progression-free survival than that of the non-smokers, with values of 0.47, 0.68, and 0.90, respectively.

Another report from a multicenter retrospective study that explored the value of oncogene driver subtype, programmed death-1 ligand (PD-L1) status, and smoking status found that smoking status potentially was the most important, easily available predictor of single PD-1/PD-L1 inhibitor efficacy.^[9]^ With a patient population of 189, the authors reported the HR value of never-smokers as 1, and that of the former and current smokers as 0.488 and 0.116 for Univariate Analysis and Multivariate Analysis, respectively.

### 1.1 Limitations of current evidence and rationale

These reports raised a very important issue: Do smoking and formerly smoking cancer patients respond better to treatment with PD-1/PD-L1 inhibitors? Along with this issue, many questions on smoking and cancer treatment remain to be answered. Our first question is whether these values represent a significant difference between the smokers and non-smokers? We also asked if any such effect is limited to this drug class. Currently five drugs are in the same PD-1/PD-L1 inhibitor category: nivolumab and pembrolizumab as the PD-1 inhibitors, and atezolizumab, durvalumab, and avelumab as PD-L1 inhibitors. The question is whether smoking status affects response to other drugs in this category in NSCLC patients. Since these drugs have also been used in treatment of other types of cancer, a further question is whether smokers and non-smokers would differ in response to treatment when these drugs are used to treat other types of cancer, and what are the differences between the drugs in PD-1/PD-L1 category and drugs of other categories (Table 1).

**Table 1:**
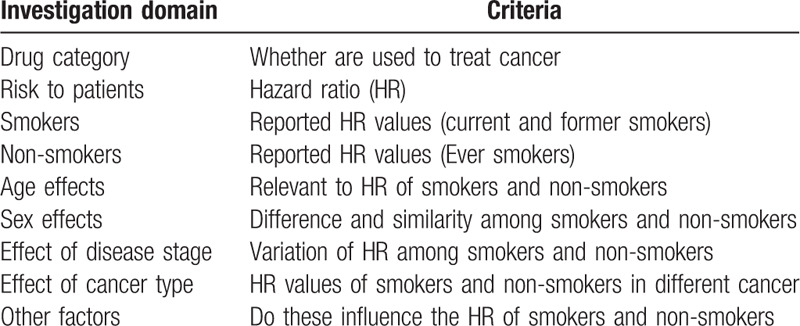
Initial approaches for evaluation of patients’ response to drug treatment.

In order to find out whether the current studies have accomplished the tasks in this protocol, on July 30, 2019, we searched PubMed (https://www.ncbi.nlm.nih.gov/pubmed/?term=smoking+cancer+treatment) for articles having the key words “smoking” and “cancer treatment” in the article title and obtained 70 publications. Among these publications, 23 were reports of smoking cessation.^[9,16]^ For example, in a retrospective cohort study, Hawari et al^[16]^ reported that smoking cessation can translate to a reduced risk of death in at least the short-term in cancer patients. While there is a general perception is that smoking cessation is beneficial to cancer treatment, only one report clearly says that smoking cessation is an essential component of treatment of lung cancer patients.^[17]^ Of 7 publications found examining the response to cancer treatment of smokers and none-smoker, 4 were published before 2008, while only 3 were published after 2015. Steinberger et al reported that cigarette smoking during external beam radiation therapy for prostate cancer is associated with an increased risk of mortality.^[18]^ Two other reports from the same study described the effect of time to simulation and treatment for patients with oropharyngeal cancer receiving definitive radiotherapy in smokers and non-smokers.^[19]^ Our search found 8 review articles on the effect of smoking during cancer treatment. While these publications suggest that smoking is a negative factor for cancer treatment, there is a lack of analyses specifically comparing the variables of drug treatment, current or former smoking status, and cancer type. None of these 70 publications reported a systematic analysis of any potential relationship between a drug and treatment of a cancer.

We next examined whether enough studies have done with PD-1/PD-L1. We searched PubMed using the key words “smoking” and “cancer treatment” and “PD-1” in the title and abstract fields. We obtained 32 publications including the report by Ng et al.^[20]^ Most of these publications are on the use of PD-1 drugs in lung cancer, in particular on PD-L1 expression as a predictable marker for suitability of treatment by drugs that inhibit PD-1/PD-L1. However, it is not clear whether smokers and non-smokers at the same level of PD-L1 expression differ in response to drug treatment.

Many clinical trials have been conducted using drugs that inhibit PD-1/PD-L1. For example, when we searched PubMed for clinical trial reports with the key words “pembrolizumab phase-III” we obtained 17 publications. When we used the key words “nivolumab phase-III,” we found 33 publications of clinical trials. Using the keywords “atezolizumab phase-III,” we obtained 74 publications. Most of these publications report results from large clinical trials. However, no systematic review and/or meta-analysis has been performed using results reported in these publications.

A most recent publication reported results from a “Systematic Review and Meta-Analysis on the Association Between Smoking and Survival Benefit of Immunotherapy in Advanced Malignancies.” However, the variable of smoking status was not analyzed in this report.^[21]^ Its result does not agree with that of Ng et al.^[20]^ Together with a few previous publications on this topic, currently no clear answer to the differences and similarities between smokers and non-smokers in response to variety of drug categories.

### 1.2 Objectives

1. To identify clinical trials that provided hazard ratio (HR) values for smoker and non-smoker participants (Table 1)
2. To collect the HR values of smokers and non-smokers and patients of subgroups, such as different age groups, sex, disease types, stages, etc
3. To collect data on drug treatment variables such as the first line treatment, second- or third-line treatments, single drug treatment, and whether treatment was combined with other chemotherapy or other drugs
4. To compare the HR values of smokers and non-smokers using ANOVA and Meta-analysis
5. To determine whether any drug works better for smokers
6. To determine whether any other factors affect the HR values of smoker vs. non-smoker cancer patients

## 2 Methods and design

**Figure 1:**
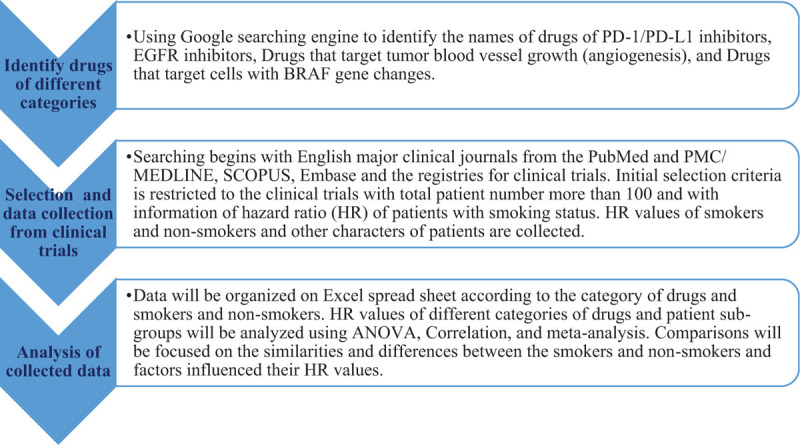
Study strategy.

Figure 1 illustrates the study strategy. Detailed items are explained in the following sections.

### 2.1 Eligibility criteria

Initially, data collection will be limited to reports of clinical trials that include

1. a total patient number (*N*) of more than 100;
2. information on smoking status of patients; and
3. results on response to treatment comparing smokers and non-smokers.

We will collect information from articles published in English published between June 30, 1900 and June 30, 2019. Literature searches will be conducted between January 15 and September 1, 2019. Late, data from studies with <100 patients will be used to confirm or support the findings from the analysis using studies with more than 100 patients.

### 2.2 Information sources

Data will be collected from major clinical trials by searching PubMed, PMC/ MEDLINE, SCOPUS, and Embase and the registries for clinical trials. The search terms for PubMed are “Drug name phase-3” and “Drug name phase-III” in the clinical trial category. The search terms for PMC/ MEDLINE are “Drug name phase-3” and “Drug name phase-III” in the title field, and “Drug name Smoking” for the abstract field. In addition, we will search the webpages of following major clinical journals: *The Lancet, The New England Journal of Medicine, The Journal of Clinical Oncology, and JAMA* (Supplementary Table 1, http://links.lww.com/MD/E874).

### 2.3 Study selection

For each publication from each database, 6 professional researchers on our team will work in pairs to screen, independently and in duplicate, titles and available abstracts to determine the eligibility of the data from the publication for inclusion in our analysis. We will acquire the full text of any publication of a clinical trial that is judged as potentially eligible by a paired review team. Two teams of reviewers will independently use the eligibility criteria to evaluate the data of potentially eligible trials. Reviewers will resolve disagreements by consensus or, if a discrepancy remains, through discussion with an arbitrator (WG or AP). The article will be excluded if the disagree still exists among arbitrators.

### 2.4 Data collection process and data items

We will use data on changes in the risk status of patients as the measurement of response to drug treatment as measured HR values, including both for progression-free survival (PFS) and overall survival (OS). Previously, we and others have used the HR of the PFS/OS ratio for measurement of drug efficacy as compared between female and male patients in clinical trials.^[7,22]^ For each article, the main text will be searched first. If the PFS or OS of patients with smoking status is not found, the supplementary materials/appendix will be searched.

Data collection will include the study drug used, last name of the first author, analytic matrix including monotherapy or combined, first line, second line, or other type of treatment, levels of PD-L1 expression, PFS and OS of patients at different smoking status (current, former, or non-smoker), *N* of patients in each smoking status category, total *N* studied, and whether the study design included randomization to treatment or not.

Data collection will include the treatment of all types of cancers treated with all types of drugs. We will start with the studies of PD-1/PD-L1 drugs, and then expand to other drugs. The key criteria are that the status of smokers and non-smoker are included in these clinical trials.

**Figure 2:**
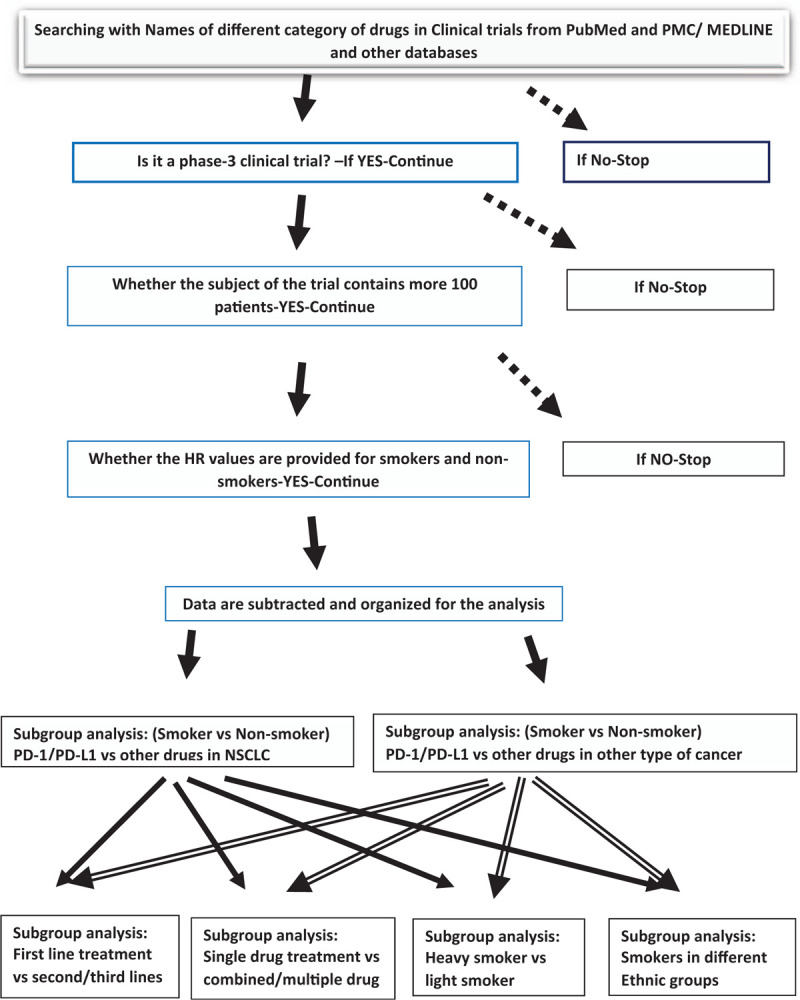
Publication searching strategy.

Data validation with corresponding authors of the studies of PD-1/PD-L1 drugs will be conducted by contacting corresponding authors of the publications included in the analysis (Fig. 2).

### 2.5 Assessment of risk of bias in individual studies

Reviewers will assess risk of bias using the Cochrane risk of bias method^[23–25]^ following the standard protocol.^[25]^ Each article will be independently reviewed by three researchers. We will evaluate the following crucial areas: randomization of participants to treatment, patient basic characterization, data collection procedures and collectors, sample sizes of smokers and non-smokers, and data analysts; other influential factors, and incomplete outcome data. Reviewers will resolve disagreement by discussion and an arbitrator (WG or BS) will adjudicate any unresolved disagreements.

Issues resolved by the risk-of-bias assessment will be taken into consideration on explanation of results of Meta-analysis. Potential influence by the risk of bias will be considered in combination with other factors. In particular, readers will be informing the potential risk of bias in the results, and these risks will be discussed when interpretation of the results.

### 2.6 Data synthesis and initial analysis

All data will be entered Excel spreadsheets. Basic statistical analysis will be conducted using analytic functional tools located in Formula Section. These tools include Student's *T* test, data, arrangement, summary, calculation of average, and correlation analysis. For *T* tests, *P* values of ≤.05were considered as statistically significant. For correlations absolute *r* values (negative or positive) were between 0.7 and 1 will be considered as significantly correlated, values of 0.35 to 0.69 will be considered as moderately correlated, while values of <0.35 will be regarded as indicating no relationship.

### 2.7 Risk of bias across studies

Specify any assessment of risk of bias that may affect the cumulative evidence (e.g., publication bias, selective reporting within studies).

### 2.8 Meta-analysis

We will conduct Meta-analysis on the data with similar procedures used in our previous publication^[7]^ and follow the PRISMA statement for reporting systematic reviews and meta-analyses.^[26]^ We will compare the results of meta-analysis with those from the *t* test.^[26]^ The means (μ vs μ) of HRs will be used to compare the two samples. Data preparation will follow our previous methods.^[7]^ Meta-analysis will be compared between smokers and non-smokers using PFS and OS.^[7,22]^

Initial data sorting and analysis will be conducted by Formulas in Microsoft Excel spreadsheet. Statistical formulas will be used to accomplish the character extraction, data alignment, data validation as well as correlation and *T* test analysis.

Open Meta-Analyst developed by Brown School of Public Health has been used to conduct a meta-analysis in this MS. Open Meta-Analyst is open-source software for performing meta-analyses of binary, continuous, or diagnostic data, using a variety fixed and random-effects methods, including Bayesian and maximum likelihood analysis. Open Meta-Analyst also enables us to conduct cumulative, leave-one-out, subgroup, and meta-regression analyses. Some features used in our study, include Continuous Fixed-Effects Inverse Variance and Continuous Random-Effects at DerSimonian-Laird methods both in Leave-One-Out analysis and in meta-analysis

### 2.9 Comparison between drugs

We will compare the effects of PD-1/PD-L1 drugs for NSCLC with other drugs including EGFR inhibitors and angiogenesis inhibitors. Both EGFR inhibitors and angiogenesis inhibitors have been used in the treatment of NSCLC. Considerable data on the response to treatment from smokers and non-smokers have been reported from a variety of clinical trials. For example, erlotinib is an EGFR inhibitor and searching PubMed using key words “erlotinib phase-III,” in the category of clinical trial, on July 31, 2019, we obtained 141 publications. Searching another EGFR inhibitor, gefitinib, in the clinical trial category resulted in 87 publications. Therefore, enough data are available for these comparisons.

Several subgroup analyses will be performed to single out the effect of PD-1/PD-L1 inhibitors on smokers and non-smokers. The effect of PD-1/PD-L1 inhibitors on NSCLC progression will also be compared to that of other types of cancer. The effect of anti-PD-1 and anti PD-L1 will be separated from that of other compounds when these drugs are combined with other drugs. Thus, we will examine separately the effect of monotherapy with PD-1/PD-L1 inhibitors with treatment by PD-1/PD-L1 inhibitors in combination with other drugs or chemotherapy.

Most importantly, we will also separate drugs used as the first line treatment from that as the second/third line or other treatment.

For comparisons between smokers and non-smokers, we will pair them in three ways: total smokers (current and former *r*) vs non-smokers, current smokers vs non-smokers, and former smokers vs non-smokers. When we compare total smokers and non-smokers, we will combine the HR values of current and former smokers into one balanced HR value using an equation of normalization: 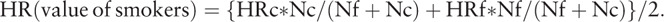


where HRc = HR values of current smokers, HRf = HR value of former smokers, Nc = number of current smokers, and Nf = number of former smokers.

In general, either PFS or OS will be provided from the studies. Some reports provided both PFS and OS or multiple PFSs from analyses of multiple sub-groups. In these cases, both PFS and OS or multiple PFSs will be analyzed with proportionally reduced population size, or divided by the number of subgroups, based on the information of subgroup analyses. 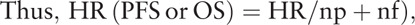


where np and nf are the number of PFS and OS given to the same subject population.

Whenever the data of more than one HR pair are included in a single study among the collected data, both continued fixed-effects and random-effects analysis models will be used. *P* values of ≤.05 will be regarded as significant in comparisons between smokers and nonsmokers. Random-effects models will be used to define a priori comparisons given the expected heterogeneity of populations from a variety of clinical trials. For each step, two persons will conduct the statistical analyses independently to confirm the results.

### 2.10 Addressing missing data

For the large clinical trials that did not provide the HR values of smokers and non-smokers, we will contact the corresponding authors to request data sharing or collaboration on the HR of patients with different smoking status. The first set of emails for data request and data confirmation will be sent on July 19 and July 20, 2019, respectively. A follow-up and/reminder email will be sent to all these corresponding authors on July 26, 2019.

### 2.11 Addressing the data balance between smokers and non-smokers in the clinical trials

As the RCTs designed to evaluate the effects of specific therapies, not the smoking status, the confounding factors would be less likely to be perfectly balanced between smokers and non-smokers. To address this issue, we will take the advantages of meta-analysis which balances the differences in the population sizes in its analysis. We will utilize the multiple models in the meta-analysis such as fixed weighted, randomized, and take-one-out of models to overcome the unbalanced data in the clinical trials.

### 2.12 Data confirmation and assessment

For data collected from each of the articles, we will send an email to the corresponding authors of the publications to confirm the values of the data. In the email, we will include

1. information on what data we have collected from the authors’ publication,
2. a request for authors to confirm the collected data and/or to add any additional information; and whether any significant factor influenced HR values of smokers and non-smokers in the study.

### 2.13 Addressing influence of other factors

To determine whether the HR values of smokers and non-smoker may be influenced by other factors, correlations will be analyzed using the HR values of smokers and non-smokers separately to compare other characteristics of patient populations. These characteristics include age, sex, ethnic groups, disease stages, pathology features, and expression levels of PD-L1, EGFR, and other mutations. If significant correlations between smoking status and other characteristics are obtained, further analysis such as meta-analysis and multiple regression analysis will be conducted.

## 3 Discussion

Our study embarks on an interesting quest to obtain new insights on potential interactions between smoking status and drug treatment in cancer patients. Tobacco contains a substantial number of chemicals that may interact with drug treatment of cancer. In some cases, these interactions have resulted in a negative impact on the efficacy of drug treatment. Therefore, smoking cessation has been the immediate primary goal for initiating cancer treatment.

While current smokers remain a large group in the general population and form most lung cancer patients, a specific drug that works well in smoking patients would be a tremendous contribution to treatment of these patients.

### 3.1 Knowledge translation

This investigation will determine whether smokers’ response to cancer treatment by any drug differs from that of non-smokers. Smoking has been considered as a major impediment to treatment of cancer patients. If smokers are found to respond to cancer treatment better than non-smokers to a category of drugs, the result would be a substantial advance over existing knowledge. It would provide information on the therapeutic application of a given drug on the smoking patient population and would potentially improve outcomes for smokers with cancer. It will open a new field of research on how smoking interacts with drug treatment, which tobacco compounds play a role in response to treatment, and how the molecular mechanism(s)is/are used in the future in drug development and cancer therapy.

This investigation will clarify whether the response of smoker and non-smoker cancer patients to drug treatment is associated with other factors, such as level of expression of PD-L1, sex, disease stage, or pathological features. If the association is identified, the results will enhance the therapeutic application of drug treatment by screening for any identified factors as potential clinical markers for the drug treatment. If such an association is not found, it suggests that effective drug treatments can be applied to both smokers and non-smokers.

### 3.2 Limitations

We may have missed some data since we only use data from PubMed, PMC/MEDLINE, SCOPUS, Embase, and the registries for clinical trials, though these literature databases are the most comprehensive data bases for medical journals. However, as we searched major clinical journals, we assumed that data from most large clinical trials were included. In addition, this review is based on data from a limited number of studies that provided the HR status of smokers and non-smokers. Since smoking status is important, future studies may need to analyze the response to treatment of patients in more discreet categories, such as current smokers, long-time smokers and new smokers, longer time former smokers, recently stopped smokers, never-smokers, and potential secondary smoke-exposed among the non-smokers.

Within the publications from PubMed, PMC/MEDLINE, SCOPUS, Embase, and the registries for clinical trials, some publications of these clinical trials did not present the data separately for smokers vs. non-smokers. Although we are attempting to contact all the primary authors for confiscation and additional information, it is unlikely that we will receive a 100% response rate. Thus, information from some of these clinical trials will likely not be included.

We realize that the population sizes of non-smokers are small in most studies. However, the small size of non-smoking populations is not only in the trials of drugs that target PD-1 and PD-L1. The small sizes exist in trials of other drugs as well. In these trials of other drugs, the non-smokers respond better than the smokers.

Overall, we are confident that these data are important for future research on the mechanism of response of smokers to certain cancer treatments and are essential not only for understanding the function of anti PD-1 and PD-L1 agents but also for the identification of new targets and development of new drugs.

## 4 Conclusions

Provide a general interpretation of the results in the context of other evidence, and implications for future research.

## 5 Authors’ information

Lishi Wang, MD, PhD, is an adjunct research professor at the University of Tennessee Health Sciences Center, Memphis, TN, USA and a full professor at Inner Mongolia Medical University, Inner Mongolia, PR China. Jing Li, MS, is a statistician at the University of Tennessee Health Science Center, Memphis, TN, USA; Helin Feng, MD PhD is a surgeon and a full professor at the Fourth Hospital of Hebei Medical University, Hebei, PR China; Feng Xia Liu, MD, MS, is a professor and the deputy director of Department of Nursing at the Fourth Hospital of Hebei Medical University, Hebei, PR China; Qingyi Liu, MD, PhD, is a surgeon, full professor, and director of the Department of First Surgery of the Fourth Hospital of Hebei Medical University, Hebei, PR China; Jiafu Ji MD, PhD, is full professor and President of Peking University Cancer Hospital and Institute, Beijing, PR China; Arnold Postlethwaite, MD, is a full professor and division chief of Rheumatology at the University of Tennessee Health Sciences Center, and Career Research Scientist at Research Service, Memphis VA Medical Center, Memphis, TN, USA; Weikuan Gu, PhD is a full professor in the Department of Orthopedic Surgery and Biomedical Engineering, the University of Tennessee Health Sciences Center, and Research Scientist at Research Service, Memphis VA Medical Center, Memphis, TN, USA; and Baoen Shan MD, PhD is a full professor and President of the Fourth Hospital of Hebei Medical University, Hebei, PR China.

## Acknowledgments

The authors thank Dr Michael G. Martin at West Cancer Center Research Institute, Memphis, TN, USA; Dr William C. Cho at Queen Elizabeth Hospital, Kowloon, Hong Kong; Dr Bernard Escudier at Gustave Roussy, Villejuif, France; Professor Kevin J. Harrington at Royal Marsden NHS Foundation Trust/The Institute of Cancer Research, London, UK; and Dr James W. Shaw at Worldwide Health Economics and Outcomes Research, Bristol-Myers Squibb, Lawrenceville, NJ, USA for their assistance and information on clinical trials. This article is written work prepared by employees of the Federal Government as part of their official duties is, under the U.S. Copyright Act, a work of the United States Government for which copyright protection under Title 17 of the United States Code is not available. We thank our professional editor, Dr Amanda Susan Clarke, at UTHSC for carefully editing our manuscript.

## Author contributions

LW and WG conceived the study design. LW, BS, and WG designed the database-specific literature search strategies. LW and JL will retrieve all potentially eligible publications. LW, HF, JL, FL, JJ, AP, QL, BS, and WG will screen potentially eligible publications and abstract data from eligible publications. AP and WG will oversee knowledge translation. AP and WG will contact investigators of clinical trials for data verifications and potential additional information. JL, HF, AP, and WG will provide oversight to ensure that our planned subgroup analyses and statistical pooling of effect sizes across patient populations, interventions, controls, and outcome measures are clinically relevant. LW and WG drafted this manuscript. All authors reviewed several drafts of the manuscript and approved the final version.

## Abbreviations

Abbreviations: HR = hazard ratio, NSCLC = non-small cell lung cancer, OS = overall survival, PD-1 = Programmed death-1, PD-L1 = Programmed death-ligand-1, PFS = progression-free survival.
